# Severe events in the first 6 months of life in a cohort of HIV-unexposed infants from South Africa: effects of low birthweight and breastfeeding status

**DOI:** 10.1111/tmi.12355

**Published:** 2014-07-23

**Authors:** Tanya Doherty, Debra Jackson, Sonja Swanevelder, Carl Lombard, Ingunn M S Engebretsen, Thorkild Tylleskär, Ameena Goga, Eva-Charlotte Ekström, David Sanders, Promise EBF study group

**Affiliations:** 1 Health Systems Research Unit, Medical Research CouncilCape Town, South Africa; 2 School of Public Health, University of the Western CapeCape Town, South Africa; 3 UNICEFNew York, USA; 4 Biostatistics Unit, Medical Research CouncilCape Town, South Africa; 5 Centre for International Health, University of BergenBergen, Norway; 6 Department of Paediatrics and Child Health, University of PretoriaSouth Africa; 7 Department of Women's and Children's Health, Uppsala UniversityUppsala, Sweden

**Keywords:** nutrition, low birthweight, hospitalisation, South Africa, breastfeeding

## Abstract

**Objective:**

To report on risk factors for severe events (hospitalisation or infant death) within the first half of infancy amongst HIV-unexposed infants in South Africa.

**Methods:**

South African data from the multisite community-based cluster-randomised trial PROMISE EBF promoting exclusive breastfeeding in three sub-Saharan countries from 2006 to 2008 were used. The South African sites were Paarl in the Western Cape Province, and Umlazi and Rietvlei in KwaZulu-Natal. This analysis included 964 HIV-negative mother–infant pairs. Data on severe events and infant feeding practices were collected at 3, 6, 12 and 24 weeks post-partum. We used a stratified extended Cox model to examine the association between the time to the severe event and covariates including birthweight, with breastfeeding status as a time-dependent covariate.

**Results:**

Seventy infants (7%) experienced a severe event. The median age at first hospitalisation was 8 weeks, and the two main reasons for hospitalisation were cough and difficult breathing followed by diarrhoea. Stopping breastfeeding before 6 months (HR 2.4; 95% CI 1.2–5.1) and low birthweight (HR 2.4; 95% CI 1.3–4.3) were found to increase the risk of a severe event, whilst maternal completion of high school education was protective (HR 0.3; 95% CI 0.1–0.7).

**Conclusions:**

A strengthened primary healthcare system incorporating promotion of breastfeeding and appropriate caring practices for low birthweight infants (such as kangaroo mother care) are critical. Given the leading reasons for hospitalisation, early administration of oral rehydration therapy and treatment of suspected pneumonia are key interventions needed to prevent hospitalisation in young infants.

## Introduction

South Africa has made insufficient progress towards achieving Millennium Development Goal (MDG) 4 to reduce under-five mortality rates by 2015, with an annual average rate of reduction between 1990 and 2012 of 1.4% (UNICEF 2013). After HIV, which is estimated to account for 28% of under-five mortality, pneumonia (12%) and diarrhoea (5%) are important contributors (Requejo *et al*. 2013). The relative contribution of pneumonia and diarrhoea is increasing as effective prevention of mother-to-child transmission (PMTCT) has resulted in a rapid decline of perinatal HIV transmission (Barron *et al*. 2013; Kerber *et al*. 2013).

Despite having a GDP per capita based on purchasing power parity (PPP) of $11 255 in 2012, compared with $2334 for sub-Saharan Africa, South Africa has substantial inequality with one of the world's highest Gini coefficients (63 *vs*. 44 for sub-Saharan Africa) (World Bank 2013). There are large gaps in coverage of certain basic child health interventions between the richest and poorest wealth quintiles. For example, coverage of vitamin A supplementation, DPT3 and measles vaccination is <20% in the poorest quintile and more than 70% in the wealthiest quintile. Similarly, coverage of oral rehydration therapy is 40% in the poorest quintile and 100% in the wealthiest (Department of Health South Africa & Measure DHS 2004).

Since 1994, there has been a free healthcare policy for pregnant women and children under the age of 6 years. However, coverage for several high-impact maternal and child health interventions remains low. Although 46% of women breastfeed within 1 h after birth, only 12% of infants are exclusively breastfed (EBF) for up to 4 months and 8% up to 6 months, one of the lowest rates on the continent with no improvement since it was first measured in 1998 (Department of Health South Africa & Measure DHS 2004). Currently, only 51% of health facilities with maternity beds are accredited as baby friendly according to WHO's *10 Steps to Successful Breastfeeding*.

Routine care during the post-natal period is low in South Africa. Post-natal care in particular needs strong links between the community and health facility to encourage healthy practices in the home, such as appropriate infant feeding and newborn care. The post-natal period is crucial for counselling about infant feeding and care seeking in order to avert early adverse events such as illness and hospitalisation, yet until recently, there was no national policy for timing or content of routine post-natal visits (Bradshaw *et al*. 2008). Furthermore, South Africa is one of a few countries in sub-Saharan Africa that does not have a national community case management programme for the treatment of pneumonia and diarrhoea by community health workers (CHWs) (UNICEF 2012).

Over the past decade, there has been a large focus on HIV in child health research in South Africa due to the contribution of HIV to under-five mortality. The PMTCT programme has achieved significant reductions in perinatal transmission from 9.6% in 2008 to 2.7% in 2011 (Barron *et al*. 2013). To achieve similar reductions in other causes of morbidity and mortality in children, research also needs to focus on HIV-unexposed children to understand modifiable or avoidable factors (Kerber *et al*. 2013).

This paper reports on factors associated with hospitalisation or death in the first 6 months of life in a cohort of HIV-unexposed infants in South Africa using data from a large multicountry community randomised trial known as PROMISE EBF (Tylleskar *et al*. 2011). The trial aimed to increase the practice of EBF through supportive counselling to mothers during home visits by community peer supporters. The intervention had effects of public health significance on EBF at 12 weeks in Burkina Faso (79.1% and 34.6%) and Uganda (81.6% and 43.9%) in the intervention and control groups, respectively (Tylleskar *et al*. 2011). In South Africa, a statistically significant increase was noted (10.5% and 6.2%; PR 1.72, 1.12–2.63); however, the public health benefit is unlikely to be significant at a population level considering the absolute change was small. In the South African context, against a background of a slow reduction in under-five mortality, it is critical to investigate factors other than HIV that are responsible for poor child health outcomes. This study collected data on feeding practices and important child health outcomes: infant hospitalisation and death. The paper will explore the associated risk factors for these severe events in the first 6 months.

## Methods

### Study design

This study used data from a community-based cluster-randomised trial promoting EBF by peer counsellors in three South African sites between 2006 and 2008 (ClinicalTrials.gov no: NCT00397150). A total of 34 clusters from three areas in South Africa were chosen: Paarl in Western Cape Province (peri-urban), and Umlazi (urban) and Rietvlei (rural) in KwaZulu-Natal Province. Infant mortality rates (IMR) and antenatal HIV prevalence at the time of the study were 40 of 1000 and 10% in Paarl, 60 of 1000 and 42% in Umlazi and 99 of 1000 and 34% in Rietvlei. The mean time to travel to the nearest hospital during the period of the study was 32 in Paarl, 84 min in Rietvlei and 35 min in Umlazi (Tlebere *et al*. 2007).

Stratified randomisation was undertaken with the strata being the three sites to avoid confounding due to known differences across the sites. A detailed description of the methodology for the trial is provided elsewhere (Tylleskar *et al*. 2011). In short, mothers in the intervention clusters received one antenatal and four post-natal visits at weeks 1, 4, 7 and 10 post-partum by peer counsellors promoting and supporting EBF for the first 6 months. In the control clusters, mothers received the same number of visits from a different group of peer counsellors who provided information to mothers about accessing social welfare grants which was not believed to have any impact on infant feeding practices nor on the main outcomes assessed in this paper.

Inclusion in the study was a two-stage process, starting with the assessment of pregnant women (pre-inclusion) and then the inclusion of the mother–infant pairs at 3 weeks post-partum (inclusion). In the last trimester of pregnancy, a total of 1148 women were approached for participation in the study from the three South African sites. At the first post-natal assessment, the mother–infant pairs were assessed and 1020 mother–infant pairs were enrolled into the PROMISE EBF study (128 were not enrolled due to relocation or being lost-to-follow-up (87), stillbirth (8), twin delivery (9) or death of the neonate (23) or mother (1) before 3 weeks after birth) (Tylleskar *et al*. 2011). Of these, 964 were HIV-unexposed and included in this subgroup analysis. The decision to focus only on HIV-negative mother–infant pairs for this analysis is due to the strong relationship between HIV infection and infant morbidity as well as feeding practices, and the HIV-positive sample was too small to analyse as a separate subgroup.

The mother–infant pairs were interviewed at recruitment late in pregnancy and at around 3, 6, 12 and 24 weeks after birth. Interview visits were regarded as timely if they were performed in the following time ranges: 3 weeks (1.5–4.5), 6 weeks (4.5–9), 12 weeks (9–18) and 24 weeks (18–28).

The recruitment interview focused mainly on socio-economic and socio-demographic characteristics. The follow-up interviews gathered self-reported data from mothers on infant feeding, morbidity, hospitalisation and mortality. Infant feeding data, including breastfeeding, were gathered using 24 h, 7 day and since birth recall of 22 specified fluid and food items. Infants were present during all follow-up visits as anthropometric measurements were also taken.

### Data management

Trained data collectors, who were kept uninformed about the allocation assignment of clusters, and kept separate from the intervention team, conducted interviews to ascertain study outcomes. Mothers' HIV status was captured from hospital records and confirmed with a laboratory-based ELISA test at 3 weeks post-partum on maternal-dried blood spots. Data were double-entered using EpiData (version 3.1).

### Event classification during follow-up

The primary outcome was occurrence of a ‘severe event’ which was defined as the composite outcome of an infant hospitalisation or death between birth and 24 weeks of age. Data on hospitalisations were self-reported at the 3,6,12, and 24 week data collection points using data from the following question ‘since birth has your child ever been hospitalised?’ Only the first hospitalisation event was considered for this analysis as repeat events were rare (three of the 57 children ever hospitalised were hospitalised twice and no child was hospitalised more than twice). The infant's actual age at first hospitalisation, death or last follow-up point (for those who survived with no hospitalisation event) was used to calculate time to the event.

### Definitions for exposure variables

The duration of breastfeeding was calculated for each infant using data from the four follow-up visits (3, 6, 12, and 24 weeks). At each follow-up visit feeding recall of 22 food and fluid items given in the last 24 h, last 7 days and since birth was collected and breastfeeding status ascertained. The midpoint between the last reported week when the infant was still breastfed and the first reported time when breastfeeding ceased was used to determine the week of stopping breastfeeding.

Birthweight was captured from the child Road to Health Chart at the 3 week post-natal data collection visit. Low birthweight was classified as a birthweight <2500 g. In the event of an infant death, the data collector was notified by the peer counsellor and performed a household visit to determine date and age at death.

### Statistical analysis

Associations between severe events and covariates were examined first using univariate analysis. An epidemiological approach was undertaken in selecting variables for the multivariate Cox proportional hazards model including demographic and socio-economic factors relevant to infant morbidity and mortality in the South African context (Victora *et al*. 1997). As early infant feeding practices are strongly associated with risk of morbidity and mortality, to estimate the effect of a change in breastfeeding status on the risk of a severe event, breastfeeding status (whether still breastfeeding or stopped) was used as a time-dependent variable in an extended Cox model. Due to the nature of the study design with sites as separate strata, a stratified extended Cox model was chosen. The final model was adjusted for study clusters using the robust cluster variance approach, as well as study arm, as the data come from a randomised trial. Estimates for hazard ratios were reported with their 95% confidence intervals. Interactions were checked and reported if significant. The proportional hazards assumption was assessed graphically and formally using Schoenfeld residuals. Statistical analyses were carried out with the use of STATA (v12) and SAS (v9.3) software.

### Ethics

Ethical approval was granted by the Ethics Committee of the Medical Research Council South Africa. Signed or thumb-printed informed consent was obtained from each mother prior to study participation.

## Results

### Characteristics of mothers and infants

Baseline characteristics of HIV-negative mother–infant pairs according to study site are summarised in Table1. The mean maternal age was 24 years in Rietvlei and Umlazi and 25 years in Paarl. A total of 10% were under the age of 18 years. Completed years of education ranged from a mean of 9 in Rietvlei to 11 in Umlazi. The majority of women in Rietvlei were married, whilst most women in Paarl and Umlazi were single. Over 90% of women in all sites had attended antenatal care. Large differences are noted in socio-economic status and access to basic infrastructure (electricity, piped water and flush toilet) between the three sites (as expected due to purposive selection), with Rietvlei being notably poorer compared with Paarl and Umlazi. The median birthweight was 3.2 kg (IQR: 2.9–3.5 kg). With regard to feeding practices, 34% (114/330), 20% (57/283) and 36% (127/351) of HIV-negative women in Paarl, Rietvlei and Umlazi, respectively, stopped breastfeeding before 6 months post-partum. The mean infant age at which breastfeeding stopped was 12 weeks in Paarl and 11 weeks in Rietvlei and Umlazi. Amongst women who stopped breastfeeding prior to 6 months, 21% did not have piped water in their house or yard.

**Table 1:** Baseline characteristics of HIV-negative mothers and infants by site

	Paarl (*n* = 330)	Rietvlei (*n* = 283)	Umlazi (*n* = 351)
Marital status			
Married	61 (18.5)	165 (58.9)	14 (3.9)
Cohabiting	11 (3.3)	0	49 (13.9)
Single/divorced/widowed/separated	258 (78.2)	115 (41.1)	288 (82.0)
Parity			
Primipara	157 (47.6)	127 (44.9)	205 (58.4)
Multipara	173 (52.4)	156 (55.1)	146 (41.6)
Attendance at antenatal clinic			
Yes	324 (98.2)	267 (94.3)	350 (99.7)
SES quintile			
1 (poorest)	0	175 (61.8)	9 (2.6)
2	35 (10.6)	79 (27.9)	62 (17.7)
3	82 (24.8)	26 (9.2)	89 (25.4)
4	119 (36.1)	3 (1.1)	89 (25.4)
5 (least poor)	94 (28.5)	0	102 (29.1)
Electricity in the house			
Yes	311 (94.2)	169 (59.7)	325 (92.6)
Water source			
Surface water and other	0	211 (74.8)	1 (0.3)
Borehole/public tap	8 (2.4)	42 (14.9)	65 (18.6)
Piped into yard or house	320 (97.6)	29 (10.3)	284 (81.1)
Type of toilet			
None/open	0	94 (38.4)	44 (12.8)
Pit/ventilated improved pit	166 (50.9)	151 (61.6)	158 (45.9)
Flush	160 (49.1)	0	142 (41.3)
Mothers age			
Mean years (SD)	25.2 (6.4)	23.8 (6.1)	23.6 (5.8)
Mothers education			
Mean years (SD)	10.0 (1.9)	8.9 (2.4)	10.9 (2.2)

Data are *n* (%) unless otherwise indicated.

### Hospitalisations

A total of 57 (6%) infants were hospitalised at least once between birth and 24 weeks of age. The median age at first hospitalisation was 8 weeks (IQR: 4–16). The main reason for hospitalisation was cough and difficult breathing (*n* = 27; 48%), followed by diarrhoea (*n* = 20; 35%). The other reasons (*n* = 10; 17%) included an accident, epilepsy, constipation and surgical problems including inguinal hernia and abscess. The majority (26/57) of hospitalised infants were from the Umlazi site.

### Infant deaths

Thirteen infants died during the follow-up period: 1 in Paarl, 4 in Rietvlei and 8 in Umlazi. The median age at death was 21 weeks (IQR 11-23). None of these infants were reported to have been hospitalised prior to the death event, so the total number of infants who experienced a severe event (either a hospitalisation or death) in the follow-up period was 70. There were no maternal deaths during the follow-up period.

### Factors associated with severe events (hospitalisations or death)

**Figure 1:**
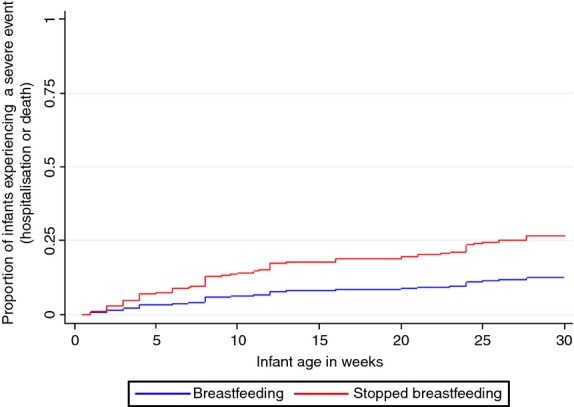
Model based failure curves of breastfeeding status. Adjusted for low birthweight, maternal education, water source, type of toilet and study arm.

Results from the univariate and multivariate Cox regression analysis are shown in Table2. In the stratified adjusted model, both breastfeeding status and low birthweight remained independently associated with a higher risk for a severe event. Completing high school was associated with a lower risk for a severe event (AHR 0.3; 95% CI 0.1–0.7). Figure1 shows adjusted modelled cumulative failure curves of breastfeeding status illustrating the increased risk for a severe event amongst infants where breastfeeding was stopped prior to 24 weeks.

**Table 2:** Stratified extended Cox regression models of hospitalisation or death, unadjusted and adjusted hazard ratios (HR) with 95% confidence intervals

	*n* (%) infants hospitalised or died between birth and 24 weeks	Unadjusted crude HR (95% CI)	Adjusted HR* (95% CI)
Study site
Paarl	23/330 (6.9)	1	
Rietvlei	13/283 (4.6)	0.7 (0.3–1.6)	
Umlazi	34/351 (9.7)	1.4 (0.8–2.5)	
Mothers education			
Some primary school	9/98 (9.2)	1	1
Some high school	46/573 (8.0)	0.8 (0.4–1.5)	0.6 (0.3–1.1)
Completed high school	12/243 (4.9)	0.5 (0.2–1.1)	0.3 (0.1–0.7)
Tertiary education	2/42 (4.8)	0.5 (0.1–2.1)	0.2 (0.0–1.0)
Household water source			
Surface water and other	9/212 (4.2)	1	1
Borehole/public tap	9/115 (7.8)	1.8 (0.5–6.7)	1.8 (0.2–12.9)
Piped into yard or house	51/633 (8.1)	1.7 (0.6–5.1)	1.6 (0.2–9.9)
Type of toilet			
Flush	27/302 (8.9)	1	1
Pit/ventilated improved pit	32/475 (6.4)	0.8 (0.5–1.3)	0.8 (0.5–1.4)
None/open	9/138 (6.5)	0.8 (0.4–1.5)	0.6 (0.3–1.6)
Study arm			
Control	33/466 (7.1)	1	1
Intervention	37/498 (7.4)	1.0 (0.6–1.8)	1.2 (0.7–2.1)
Low birthweight			
No	60/836 (7.2)	1	1
Yes	8/54 (14.8)	2.2 (1.2–4.0)	2.4 (1.3–4.3)
Breastfeeding (BF) status†			
Still breastfeeding at 24 weeks	47/677 (6.9)	1	1
Stopped breastfeeding before 24 weeks	23/287 (8.0)	2.7 (1.4–4.9)	2.4 (1.2–5.1)

* Stratified by site.

† Time-dependent covariate.

## Discussion

The study found that amongst HIV-unexposed infants, low birthweight and a short duration of breastfeeding increased the risk for hospitalisation or death in the first 6 months of life, whilst completion of high school education was protective. The median age at first hospitalisation was 8 weeks which could explain why low birthweight was such a strong risk factor. Although South Africa has a low birthweight prevalence of only 8% (Department of Health South Africa & Measure DHS 2004), low birthweight was ranked the fifth leading cause of death in children under five in 2007 (Nannan *et al*. 2012). Sanitation and water source were not found to be associated with severe events in this context underscoring the risks posed by lack of breastfeeding even in a relatively well-resourced African setting.

The largest number of hospitalisations occurred in the urban Umlazi site. This site is well resourced in terms of access to clinics and a referral hospital and recent research has reported that 56% of mothers in this area can walk to referral facilities (Nsibande *et al*. 2013). In comparison, the average time to reach a hospital in the rural Rietvlei site is 84 min (Tlebere *et al*. 2007). A study describing pathways to care seeking during fatal infant illness in Rietvlei and Umlazi found that home remedies were very common in Rietvlei and only half of the mothers interviewed had taken their infant to a public hospital during the period of illness. The study reported that mothers perceived the quality of care at clinics to be poor (Sharkey *et al*. 2012). Umlazi was also the site with the highest proportion of women who stopped breastfeeding before 6 months (36% compared with 20% in Rietvlei).

In terms of prevention, there is a need to address potential contributing factors to low birthweight in South Africa, particularly adolescent pregnancy, maternal infections and, in certain regions, substance abuse (Olivier *et al*. 2013). Pre-natal and antenatal interventions are needed to encourage completion of high school, reduce adolescent pregnancy and substance abuse in young women. A cluster-randomised trial is underway in South Africa assessing the impact of cash incentives given to high school learners on several outcomes including rates of pregnancy, contraceptive use, substance use and academic performance (Abdool Karim 2012). There is also a need to scale up simple interventions known to improve breastfeeding, health and survival of low birthweight babies, such as kangaroo mother care (Lawn *et al*. 2010; March of Dimes *et al*. 2012).

The relationship between lack of breastfeeding and morbidity in low HIV prevalent developing countries has been well described (WHO Collaborative Study Team on the Role of Breastfeeding on the Prevention of Infant Mortality 2000; Bahl *et al*. 2005). Data from a multicentre cohort study conducted in India, Ghana and Peru found a threefold increased risk of all cause hospitalisation in the first 6 months of life amongst non-breastfed infants compared with predominantly breastfed infants (Bahl *et al*. 2005). A major difference between this study and ours is that at 22 weeks 85% of infants in these three countries were still being breastfed, whilst in our study, 31% of mothers stopped breastfeeding before 24 weeks (median infant age 11.5 weeks). Our study highlights the risk posed by non-breastfeeding even in an upper middle-income country (South Africa). The most common reasons for hospitalisation in our study were pneumonia and diarrhoea, causes known to be associated with lack of breastfeeding (WHO Collaborative Study Team on the Role of Breastfeeding on the Prevention of Infant Mortality 2000).

In South Africa, prior to 2011 the policy environment was not supportive of active breastfeeding promotion. Given the high antenatal HIV prevalence, the guidelines and policies to prevent mother-to-child transmission of HIV had focussed on limiting or avoiding exposure to breast milk either through provision of free formula milk or through shorter duration of breastfeeding (Doherty *et al*. 2011). The lack of active promotion of breastfeeding in South Africa has resulted in no improvement in breastfeeding practices, which were already poor prior to the emergence of the HIV epidemic (Department of Health/Medical Research Council/Measure DHS 1998). Following changes in the WHO guidance regarding infant feeding in the context of HIV in late 2010, with a shift towards promotion of exclusive breastfeeding with antiretroviral prophylaxis, (World Health Organisation 2010) South Africa made several policy changes including developing a new Infant and Young Child Feeding Policy with promotion of breastfeeding as a key goal, legally enforcing the Code of Marketing of Breastmilk Substitutes and a decision to stop distribution of free formula milk for HIV-positive women. These policy changes will hopefully lead to an era where breastfeeding can be promoted to all women through clear and consistent messages at community and health facility levels.

Improving post-natal care, especially community-based care and promotion of breastfeeding, is necessary in order to intervene early to prevent severe events such as hospitalisation (Imdad & Bhutta 2013). The South African government is currently revitalising the health system with a much greater focus on primary health care including the addition of ward-based outreach services using CHWs (South African National Department of Health 2010). Despite the provision of home-based peer counsellor visits to mothers in the intervention arm of the PROMISE EBF trial, this analysis has shown that short duration of breastfeeding was common, even amongst HIV-negative women. Perhaps the focus on exclusivity of breastfeeding and not duration of breastfeeding could have contributed to the low overall impact of the intervention. Very little attention has been paid to duration of breastfeeding in current South African policies which promote exclusive breastfeeding. This aspect of policy and guidelines is often overlooked as more attention is paid to the pattern of breastfeeding, especially exclusivity.

In terms of treatment, there is now substantial experience of, and considerable evidence for, the effectiveness of community management of childhood infections such as pneumonia and diarrhoea (Sazawal & Black 2003; Dawson *et al*. 2008; Chopra *et al*. 2013; WHO/UNICEF 2013). Although a national CHW programme has recently been initiated in South Africa, community management of these infections is not yet part of their job description. The findings of this study provide evidence to support an initiative on community case management to reduce the likelihood of hospitalisation and mortality from these conditions.

Our study had several potential limitations. The sites where this research was undertaken were purposely selected, and the infrastructural conditions differed greatly between them. They do, however, reflect the range of typical settings in South Africa (urban, peri-urban or rural). There may be other factors associated with infant hospitalisation in our sample that we did not measure, such as maternal health status. Recall bias could have been introduced due to the fairly long periods between data collection visits, especially between the last two visits (12–24 weeks). However, hospitalisation of an infant can be considered a major family event, and the likelihood of recall bias with this outcome is small. We were unable to distinguish the causes of low birthweight (pre-maturity *vs*. intra-uterine growth restriction) which can have differing health effects, due to the lack of reliable gestational age at birth data from delivery records.

The study also has several strengths. The sample was large, and data on infant feeding and hospitalisation were rigorously collected at four points during the first half of infancy. Data on hospitalisations were collected as part of a community-based, not a hospital-based study.

## Conclusion

A strengthened primary healthcare system incorporating promotion of breastfeeding and appropriate caring practices for low birthweight infants (such as kangaroo mother care) are critical. Given the leading reasons for hospitalisation, early and correct administration of oral rehydration therapy and recognition and early treatment of suspected pneumonia are key interventions needed to prevent hospitalisation events in young infants. This paper re-emphasises that even within an upper middle-income country, such as South Africa, continued breastfeeding beyond the first few months of life is crucial for optimising child health and survival.

## Appendix

### The Promise-EBF study group

Steering Committee: Thorkild Tylleskär, Philippe Van de Perre, Eva-Charlotte Ekström, Nicolas Meda, James K. Tumwine, Chipepo Kankasa, Debra Jackson. Participating countries and investigators: Norway: Thorkild Tylleskär, Ingunn M. S. Engebretsen, Lars T. Fadnes, Eli Fjeld Falnes, Knut Fylkesnes, Jørn Klungsøyr, Anne Nordrehaug-Åstrøm, Oystein Evjen Olsen, Bjarne Robberstad, Halvor Sommerfelt; France: Philippe Van de Perre; Sweden: Eva-Charlotte Ekström, Barni Nor; Burkina Faso: Nicolas Meda, Abdoulaye Hama Diallo, Thomas Ouedrago, Jeremi Rouamba, Bernadette Traoré, Germain Traoré, Emmanuel Zabsonré; Uganda: James K. Tumwine, Caleb Bwengye, Charles Karamagi, Victoria Nankabirwa, Jolly Nankunda, Grace Ndeezi, Margaret Wandera; Zambia: Chipepo Kankasa, Mary Katepa-Bwalya, Chafye Siuluta, Seter Siziya; South Africa: Debra Jackson, Mickey Chopra, Mark Colvin, Tanya Doherty, Ameena E Goga, Carl Lombard, Lungiswa Nkonki, David Sanders, Rebecca Shanmugan, Wesley Solomons, Wanga Zembe; Country PI first, others in alphabetical order of surname.
